# Downregulation of p53 drives autophagy during human trophoblast differentiation

**DOI:** 10.1007/s00018-017-2695-6

**Published:** 2017-10-27

**Authors:** Martin Gauster, Sabine Maninger, Monika Siwetz, Alexander Deutsch, Amin El-Heliebi, Dagmar Kolb-Lenz, Ursula Hiden, Gernot Desoye, Florian Herse, Andreas Prokesch

**Affiliations:** 1 Institute of Cell Biology, Histology and Embryology, Medical University Graz, Neue Stiftingtalstraße 6, F/03/38, 8010 Graz, Austria; 2 Division of Hematology, Department of Internal Medicine, Medical University Graz, Graz, Austria; 3 Center for Medical Research, Core Facility Ultrastructure Analysis, Medical University Graz, Graz, Austria; 4 Department of Obstetrics and Gynecology, Medical University Graz, Graz, Austria; 5 Experimental and Clinical Research Center, A Joint Cooperation Between the Charité Medical Faculty and the Max-Delbrueck Center for Molecular Medicine, Berlin, Germany; 6 Berlin Institute of Health (BIH), Berlin, Germany

**Keywords:** Pregnancy, Placenta, Development, Trophoblast fusion

## Abstract

The placental barrier is crucial for the supply of nutrients and oxygen to the developing fetus and is maintained by differentiation and fusion of mononucleated cytotrophoblasts into the syncytiotrophoblast, a process only partially understood. Here transcriptome and pathway analyses during differentiation and fusion of cultured trophoblasts yielded p53 signaling as negative upstream regulator and indicated an upregulation of autophagy-related genes. We further showed p53 mRNA and protein levels decreased during trophoblast differentiation. Reciprocally, autophagic flux increased and cytoplasmic LC3B-GFP puncta became more abundant, indicating enhanced autophagic activity. In line, in human first trimester placenta p53 protein mainly localized to the cytotrophoblast, while autophagy marker LC3B as well as late autophagic compartments were predominantly detectable in the syncytiotrophoblast. Importantly, ectopic overexpression of p53 reduced levels of LC3B-II, supporting a negative regulatory role on autophagy in differentiating trophoblasts. This was also shown in primary trophoblasts and human first trimester placental explants, where pharmacological stabilization of p53 decreased LC3B-II levels. In summary our data suggest that differentiation-dependent downregulation of p53 is a prerequisite for activating autophagy in the syncytiotrophoblast.

## Introduction

The placenta is responsible for pregnancy-maintaining functions, such as exchange of gases and metabolites, secretion of essential hormones, and regulation of water balance. Placental villi are covered by a continuous layer of a multinucleated trophoblast, referred to as syncytiotrophoblast, and underlying mononucleated cytotrophoblasts. Together they form a major part of the placental barrier, which is maintained by a unique cellular turnover: a small subset of mitotically active progenitors among the cytotrophoblast population divides in an asymmetric way, generating one daughter cell with a preserved proliferation-competent status and a second that escapes the cell cycle. These post-mitotic cells undergo differentiation and finally fuse with the syncytiotrophoblast. Trophoblast differentiation and fusion are regulated by multiple factors, including cytokines, hormones, protein kinases, transcription factors, proteases, and membrane proteins [17], although most of these factors are in fact not bona fide fusogens [16]. Upon fusion, isolated short fragments of plasma membrane can be observed within the syncytioplasm and are presumably recycled in due time [4].

One mechanism participating in disassembly of residual plasma membrane and in cytoskeleton rearrangement in trophoblast syncytialization can be macroautophagy (hereafter referred to as autophagy). As a cell survival process generally important for degradation of aggregates of misfolded proteins or damaged organelles, autophagy is responsible for intracellular ‘revitalization’, which seems to be particularly important for the maintenance of terminally differentiated cells [34]. In the context of remodeling and differentiation, the catabolic degradation of cellular components represents a crucial source of metabolites and precursor molecules required in anabolic processes [33]. Accordingly, autophagy is ascribed fundamental roles in the differentiation of various cell types, including hematopoietic cells [41], myoblasts [14], lens epithelial cells [3], and epidermal keratinocytes [2]. In human placenta, autophagy is described to protect the syncytiotrophoblast from apoptosis [53], bacterial infection [8], and inflammation [58]. Although some signaling hubs, such as mechanistic target of rapamycin (mTOR) and 5′ adenosine monophosphate-activated protein kinase (AMPK) are well described in regulating (placental) autophagy, many questions still remain regarding precise mechanisms ensuring autophagic flux in the course of human trophoblast differentiation and fusion.

Here, the trophoblast cell line BeWo was used to study expression of autophagy-related genes and their upstream regulators during trophoblast differentiation. Transcriptome analyses showed considerable upregulation of autophagy-relevant genes in differentiated trophoblasts and identified p53 as a putative negative upstream regulator. Based on these initial observations, the hypothesis whether p53 regulates autophagy in human trophoblast differentiation was addressed in several experimental systems.

## Results

### Global transcriptome characterization of BeWo differentiation

**Fig. 1:**
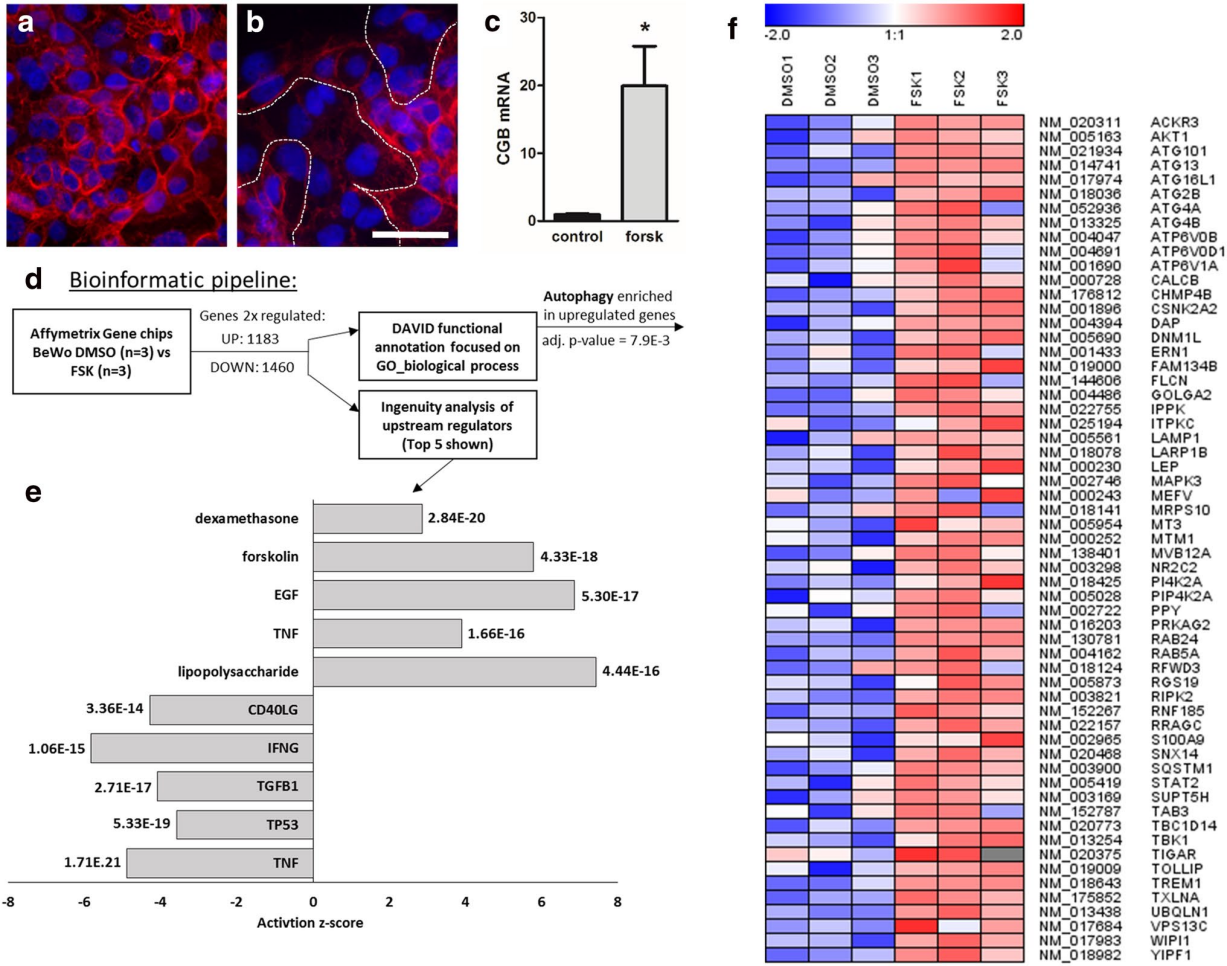
Transcriptome analyses of BeWo cell differentiation. Immunofluorescence staining of E-cadherin in BeWo cells incubated either with vehicle control (**a**, DMSO, 0.1%) or forskolin (**b**, 20 µM). Stimulation with forskolin induced formation of multinucleated syncytia (outlined by dotted lines) after 48 h culture. Scale bar in (**b**) represents 100 µm. **c** qPCR analysis showed significantly upregulated mRNA expression of β subunit of human chorionic gonadotropin (CGB) in forskolin-treated BeWo cells after 48 h. Data are presented as mean ± SEM *p ≤ 0.05. **d** Bioinformatic pipeline applied to twofold regulated genes is depicted. **e** Top five positive (positive *z*-score) and negative (negative *z*-score) upstream regulators derived from ingenuity pathway analysis (IPA), sorted by adjusted *p* values given next to bars. **f** Heatmap of genes comprising the gene ontology (GO) term “autophagy” that is significantly enriched (adjusted *p* value = 7.9E−3) in a DAVID functional annotation analysis focused on GO biological processes. Data are from three independent experiments, using different cell passages

The trophoblast cell line BeWo was used as in vitro model to study global transcriptome changes in villous trophoblast differentiation. For this purpose, BeWo cells were cultured for 48 h in the presence or absence of forskolin, a compound well-described to induce syncytiotrophoblast formation [54]. First, syncytialization and endocrine differentiation of forskolin-stimulated BeWo cells were confirmed by immunofluorescence staining of E-cadherin and by measuring of mRNA expression of β subunit of human chorionic gonadotropin (CGB). E-cadherin staining showed that vehicle control cells remained mononucleated (Fig. 1a), whereas forskolin induced pronounced formation of multinucleated syncytia (Fig. 1b). Syncytialization was accompanied by a 20.7-fold increase in CGB expression, when compared to vehicle control (Fig. 1c). After having confirmed differentiation and syncytialization, mRNA from forskolin-stimulated and vehicle control BeWo cells was subjected to differential microarray analysis and controls. This yielded to 1183 upregulated and 1460 downregulated genes that are at least changed twofold. Top upregulated genes contain marker genes of ‘*syncytiotrophoblastogenesis*’, such as several isoforms of CGB [31], or placental growth factor (PGF) [12], verifying the plausibility of our data. To analyze this data set on pathway and gene ontology (GO) levels, we used ingenuity pathway analysis (IPA) and DAVID functional annotation, respectively (Fig. 1d). IPA includes data-based prediction of potential upstream regulators. The top five regulators with a positive and a negative activation *z*-score are shown in Fig. 1e.

Consistent with the treatment applied, forskolin was identified as highly significant activating regulator. Other regulators with positive *z*-score were epidermal growth factor (EGF), TNF, lipopolysaccharide (LPS), and dexamethasone, all of which were reported to participate in placenta development or fusion in other cell systems [23, 36, 50, 56]. Interestingly, p53 was among the top negative regulators (Fig. 1e). While the expression of p53 has been investigated in placental samples [19, 21, 44] and in the context of trophoblast invasion [35, 49], it has not been described as regulator in villous trophoblast differentiation. Besides being a prominent tumor suppressor in cancer cells, p53 is known to play a role in autophagy regulation [30]. Consistently, performing DAVID functional annotation on the upregulated genes yielded the GO biological process “autophagy” (GO:0006914) as one of many significantly overrepresented GO terms. The expression levels of genes contained in the autophagy GO term are depicted as heat map in Fig. 1f, showing upregulation of many autophagy-related genes (ATGs), as well as autophagosomal (*SQSTM1*, also known as p62) and lysosomal (*LAMP1*) markers. In summary, our analyses revealed autophagy as upregulated during trophoblast differentiation, while p53 appeared as a negative regulator of this process.

### p53 is downregulated during trophoblast differentiation

**Fig. 2:**
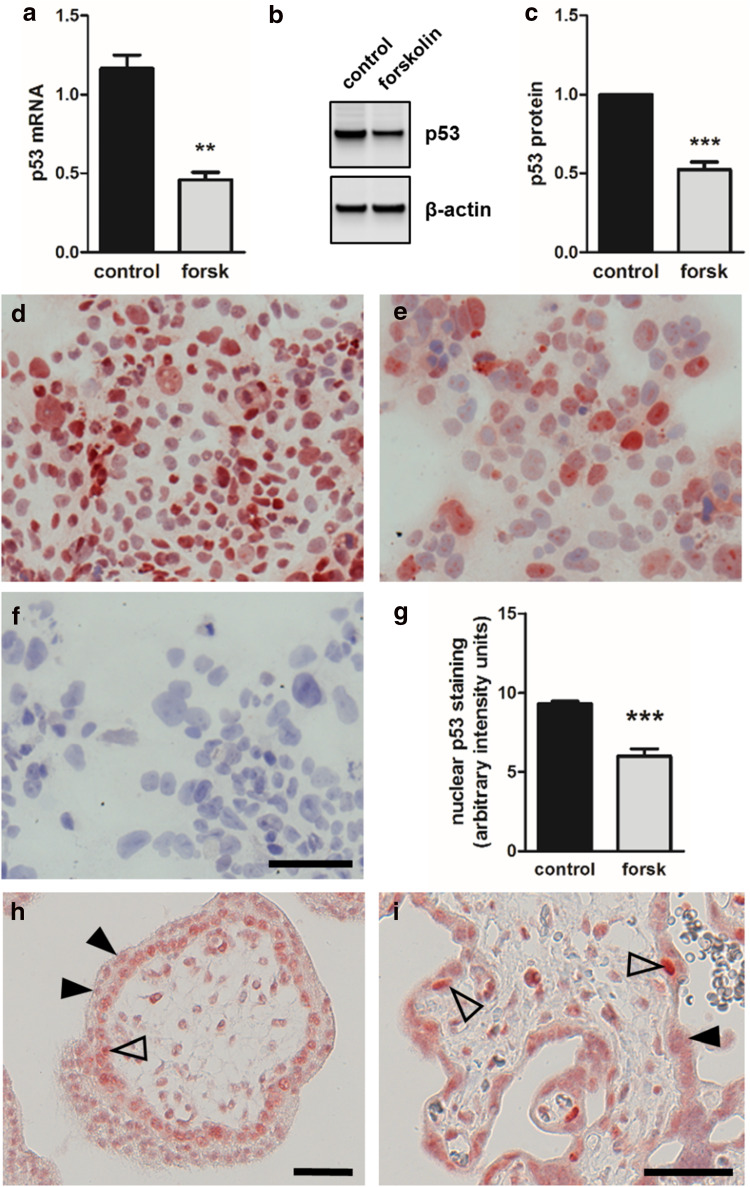
p53 expression in BeWo differentiation and human villous placenta. qPCR analysis (**a**) and immunoblotting (**b**) with subsequent band densitometry (**c**), showed significantly downregulated p53 mRNA and protein, respectively, in forskolin treated (20 µM) BeWo cells, compared to vehicle control (DMSO, 0.1%) after 48 h. Immunocytochemistry of BeWo cells treated with vehicle only (DMSO, 0.1%) detected p53 in a considerable proportion of nuclei (**d**), while stimulation with forskolin (20 µM, 48 h) decreased the amount of positive stained nuclei (**e**). Incubation with negative control mouse IgG2a yielded no staining in BeWo (**f**). Nuclear p53 staining was quantified using a cell image analysis software (**g**). Immunohistochemistry of human first trimester (**h**) and term placenta (**i**) located p53 in nuclei of cytotrophoblasts (open arrowheads), whereas nuclei within the syncytiotrophoblast showed only scant staining (arrowheads). Scale bar in **f** represents 100 µm and in **h** and **i** 50 µm. Data in **a** and **c** are presented as mean ± SEM from four and six independent experiments, respectively, using different cell passages. **p ≤ 0.01, ***p < 0.001

We further scrutinized the role of p53 as negative regulator during trophoblast differentiation and syncytialization. Forskolin-induced differentiation of BeWo cells was accompanied with a 2.9-fold decrease of p53 mRNA levels, when compared to vehicle control after 48 h culture (Fig. 2a). In line with mRNA data, p53 protein declined by 47.6% in forskolin-treated BeWo cells, compared to control (Fig. 2b, c). Immunocytochemical staining of non-stimulated BeWo cells, i.e., cells incubated with vehicle control alone, showed abundant p53 staining in the majority of nuclei (Fig. 2d). However, when cells were cultured in the presence of forskolin, the number of p53 positive nuclei significantly declined by 35.6% (Fig. 2e, g).

Our observations in BeWo culture reflected the in situ situation, as determined by immunohistochemistry of human first trimester and term villous placental tissue. In first trimester placental villi, p53 was detected in nuclei of most villous cytotrophoblasts, whereas nuclei in the overlying syncytiotrophoblast showed only weak to no staining (Fig. 2h). This pattern was also detected in term placenta, showing strong p53 staining in the rare cytotrophoblast population and only scant staining in the syncytiotrophoblast (Fig. 2i).

### Autophagy markers p62 and LC3B-II increase during trophoblast differentiation

**Fig. 3:**
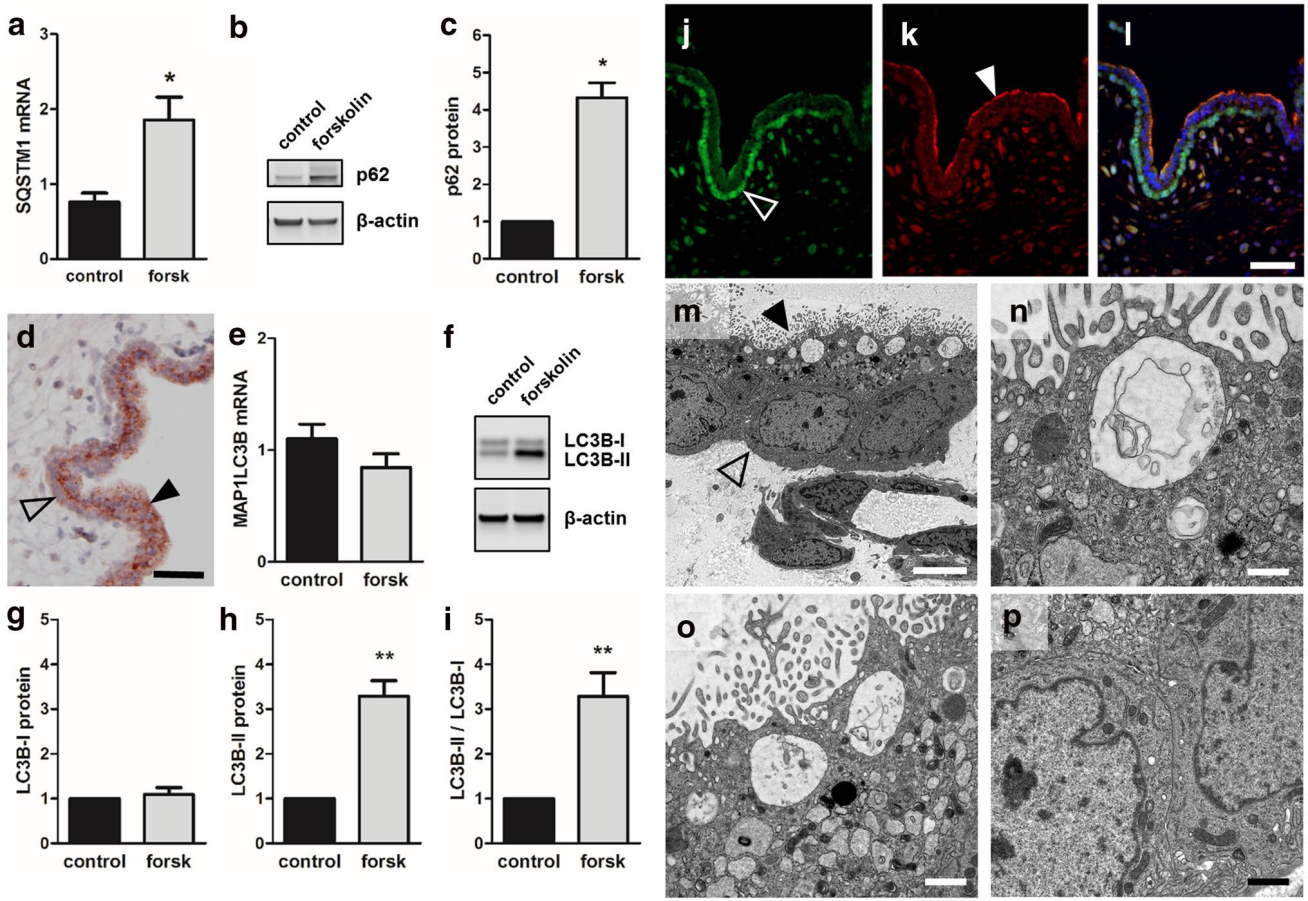
p62 and LC3B expression in BeWo differentiation and human villous placenta. qPCR analysis of p62 mRNA (**a**, SQSTM1), immunoblotting (**b**) and band densitometry (**c**) showed significantly increased p62 levels after 48 h incubation with forskolin (20 µM), when compared to vehicle control (DMSO, 0.1%). Immunohistochemistry of human first trimester placenta (**d**) located p62 in the syncytiotrophoblast (arrowhead), whereas cytotrophoblasts (open arrowhead) showed only weak staining. qPCR analysis of LC3B mRNA (MAP1LC3B) did not show significant differences between forskolin-treated (20 µM) BeWo cells and vehicle control after 48 h (**e**). Immunoblotting (**f**) and band densitometry revealed no difference in LC3B-I levels (**g**), whereas LC3B-II levels (**h**) and the ratio of LC3B-II to LC3B-I (**i**) significantly increased in response to forskolin (20 µM, 48 h). Immunofluorescence double staining showed p53 staining in nuclei of villous cytotrophoblasts (**j**, open arrowhead), while LC3 staining was predominantly detected in apical regions of the syncytiotrophoblast (**k**, arrowhead). Merge of p53 and LC3 immunofluorescence is shown in (**l**). Transmission electron microscopy (TEM) analysis of human first trimester villous cytotrophoblast (**m,** open arrowhead) and syncytiotrophoblast (**m**, arrowhead) showed late autophagic compartments underneath the microvillous plasma membrane of the syncytiotrophoblast (**n**, **o**), whereas the cytoplasm of villous cytotrophoblasts (**p**) were devoid of such structures. Scale bars in **d** and **l** represent 50 µm. Scale bar in **m** represents 5000 nm and those in **n**–**p** 1000 nm. Data in **a**–**e** and those in **f**–**i** are presented as mean ± SEM from three and six independent experiments, respectively, using different cell passages. *p ≤ 0.05, **p ≤ 0.01

To test whether trophoblast differentiation is accompanied by induction of autophagy as suggested by our transcriptome analyses, the effects of forskolin stimulation on autophagy markers p62 and LC3B were analyzed in BeWo cells and human first trimester placenta. Accordingly, forskolin treatment of BeWo cells showed 1.9- and 4.3-fold increased p62 mRNA (Fig. 3a) and protein levels (Fig. 3b, c), respectively, when compared to control. Also, immunohistochemistry of human first trimester placenta revealed a distinct, spotted staining pattern for p62 in the syncytiotrophoblast, whereas the villous cytotrophoblast layer showed only a pale homogeneous staining (Fig. 3d).

While p62 upregulation confirmed our microarray data (Fig. 1f), we used LC3 as autophagy marker in further analyses. This was informed by the state-of-the-art autophagy guidelines [26] that also establish lipidation of endogenous LC3B via western blot, in combination with autophagy inhibitors, as the most reliable indicator of autophagosome turnover (i.e. autophagic flux). Gene expression analysis of the LC3B gene *MAP1LC3B* did not show any significant deregulation in response to forskolin after 48 h incubation (Fig. 3e). However, on protein level, the posttranslationally processed variant LC3B-II, which undergoes lipidation with phosphatidylethanolamine and binds to the outer membrane of autophagosomes, significantly increased 3.3-fold, while non-lipidated cytosolic LC3B-I remained unchanged, when compared to vehicle control (Fig. 3f–h). Thus, the ratio of LC3B-II to LC3B-I significantly increased during forskolin-induced BeWo differentiation (Fig. 3i). Immunofluorescence double staining of human first trimester placenta showed a reciprocal staining pattern of p53 and LC3 in the double-layered villous trophoblast compartment. While p53 was predominantly located in nuclei of villous cytotrophoblasts (Fig. 3j), LC3 was detected in the apical cytoplasmic part of the syncytiotrophoblast (Fig. 3k). Apical LC3 localization in the syncytiotrophoblast was in good agreement with subsequent transmission electron microscopy (TEM) analysis of human first trimester placenta (Fig. 3m), showing structures reminiscent of late autophagic compartments, i.e., late autophagolysosomes, directly underneath the microvillous plasma membrane of the syncytiotrophoblast (Fig. 3n, o), whereas the cytoplasm of villous cytotrophoblasts was devoid of such structures (Fig. 3p).

### Autophagic activity is enhanced during trophoblast differentiation

**Fig. 4:**
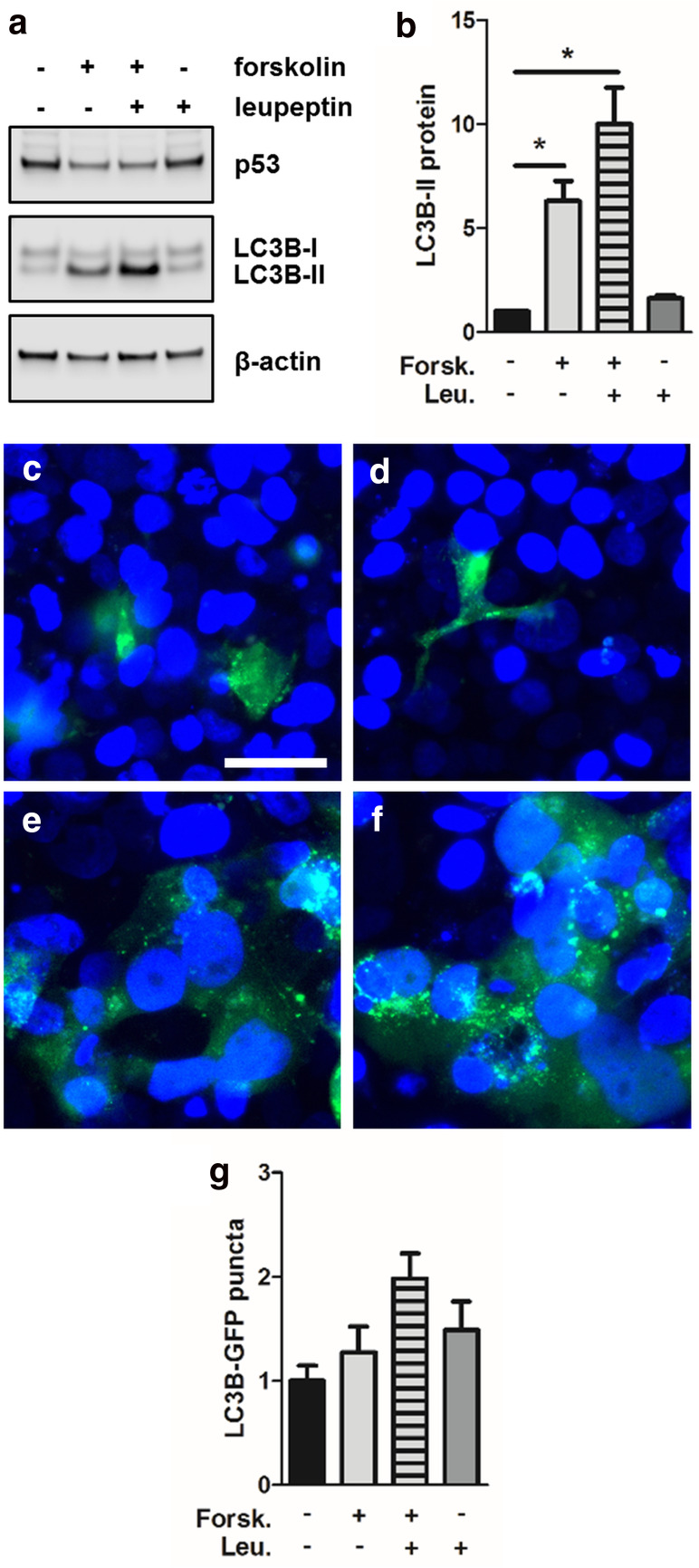
Autophagic activity is increased in BeWo differentiation. Immunoblotting (**a**) and band densitometry (**b**) showed that leupeptin (100 µM) treatment further augmented forskolin-induced (20 µM, 48 h) increase of LC3B-II levels, when compared to forskolin treatment alone. Transfection of BeWo cells with a LC3B-GFP construct showed moderate LC3B puncta in vehicle control (**c**, DMSO, 0.1%), which increased after leupeptin (100 µM) treatment (**d**). Forskolin stimulation (**e**) increased LC3B puncta compared to vehicle control, and further increased after both forskolin and leupeptin administration (**f**). Software-based image analysis of LC3B-GFP puncta (**g**) by trend confirmed data obtained with immunoblotting. Scale bar in **c** represents 50 µm. Data in **b** are presented as mean ± SEM from three independent experiments, using different cell passages. *p ≤ 0.05

The fact that LC3B-II levels significantly increased in BeWo cells upon forskolin stimulation, while LC3B gene expression remained stable, indicates either a blockage of autophagy (thereby leading to accumulation of LC3B-II) or increased autophagic activity during trophoblast differentiation [26]. Autophagic activity (or flux) is usually determined by measuring cellular LC3B-II turnover in the presence or absence of leupeptin, a membrane-permeable thiol protease inhibitor that blocks autophagy by impairing amphisome–lysosome fusion [20]. Since lysosomes are the dominant site of LC3B-II clearance, leupeptin administration mediates LC3B-II accumulation through attenuated lysosomal decay. In BeWo cells, leupeptin administration further augmented forskolin-induced increase of LC3B-II levels (Fig. 4a), which were increased by 59% compared to forskolin treatment alone (Fig. 4b). Interestingly, leupeptin per se increased low LC3B-II levels in vehicle control cells by 63%, which did not reach statistical significance, but suggested some extent of basal autophagic activity in non-stimulated BeWo cells (Fig. 4a, b).

These observations were confirmed by transfection of BeWo cells with a LC3B-GFP construct, generating green cytoplasmic LC3B puncta depending on the stimulation and degree of autophagic activity. In line with LC3B-II immunoblot data, control BeWo cells showed moderate levels of LC3B puncta (Fig. 4c), which increased after leupeptin treatment (Fig. 4d). However, when cells were stimulated with forskolin, LC3B puncta increased compared to vehicle control (Fig. 4e), and further increased after both forskolin and leupeptin administration (Fig. 4f), as determined by software-based image segmentation and pixel counting (Fig. 4g).

### Prevention of p53 decrease reduces autophagic activity in trophoblast differentiation

**Fig. 5:**
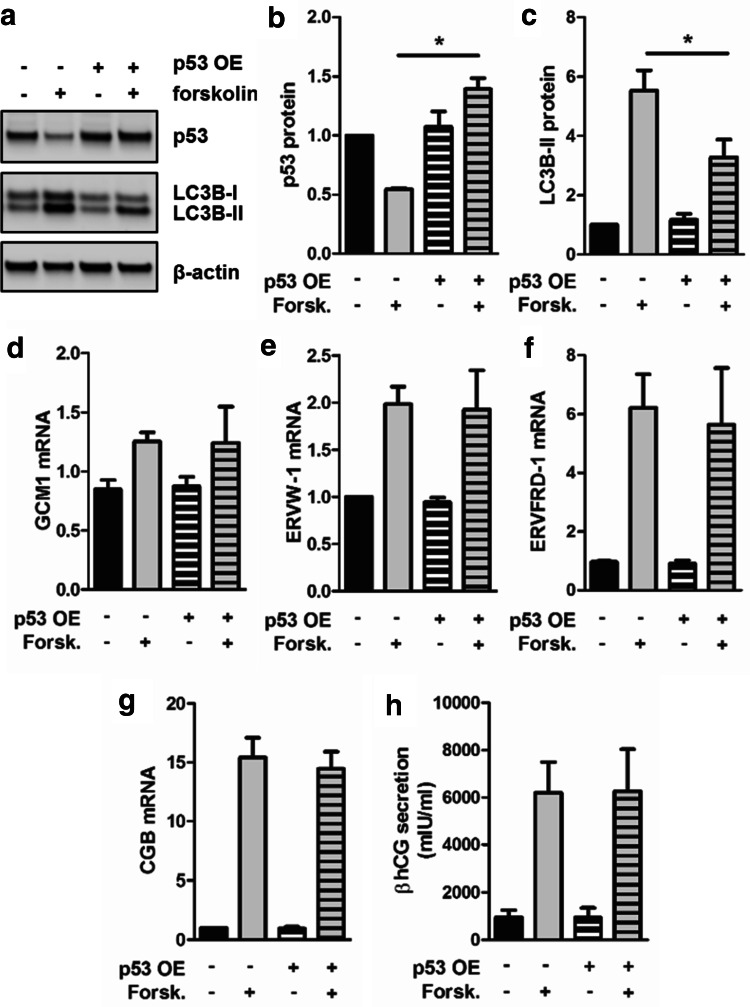
p53 overexpression reduced autophagic activity in BeWo differentiation. BeWo cells were transfected either with a p53 expressing vector (+p53 OE) or empty vector control (−p53 OE) and were subsequently incubated with forskolin (20 µM) or vehicle control (DMSO, 0.1%), respectively, for 48 h. Immunoblot (**a**) and band densitometry data for p53 (**b**) and LC3B-II (**c**) levels showed that p53 overexpression abrogated forskolin-mediated downregulation of endogenous p53 and at the same time significantly reduced LC3B-II levels compared to forskolin-stimulated empty vector control. p53 overexpression did not affect forskolin-induced expression of GCM1 (**d**), syncytin-1 (**e**, ERVW-1), syncytin-2 (**f**, ERVFRD-1), as well as expression (**g**, CGB) and secretion (**h**) of hCG-beta subunit. Data in **b**–**h** are presented as mean ± SEM from three independent experiments, using different cell passages. Significant differences between p53 overexpression (+p53 OE) and empty vector control (−p53 OE) in forskolin-stimulated cells are indicated *p ≤ 0.05

To test whether downregulation of p53 is connected to increased autophagy in trophoblasts, we aimed to retain p53 levels during differentiation by ectopic expression. While transfection with empty vector and subsequent forskolin treatment again showed significant downregulation of p53 protein, p53 overexpression completely abrogated forskolin-induced downregulation of p53 and showed no significant difference in p53 protein levels between forskolin stimulation and vehicle control (Fig. 5a, b). However, overexpression of p53 significantly reduced LC3B-II levels in forskolin-stimulated BeWo cells by 40.7%, when compared to forskolin-treated empty vector control (Fig. 5c). Interestingly, markers of villous trophoblast differentiation, such as expression of transcription factor GCM1 (Fig. 5d) and its downstream targets syncytin-1 (ERVW-1, Fig. 5e) and syncytin-2 (ERVFRD-1, Fig. 5f) as well as expression and secretion of human chorionic gonadotropin (hCG) beta-subunit (Fig. 5g, h), were not affected by ectopic overexpression of p53—neither in forskolin-stimulated cells nor in controls. These data suggested that activation of autophagy is specifically blocked by circumvention of p53 downregulation, while trophoblast differentiation is unaffected.

**Fig. 6:**
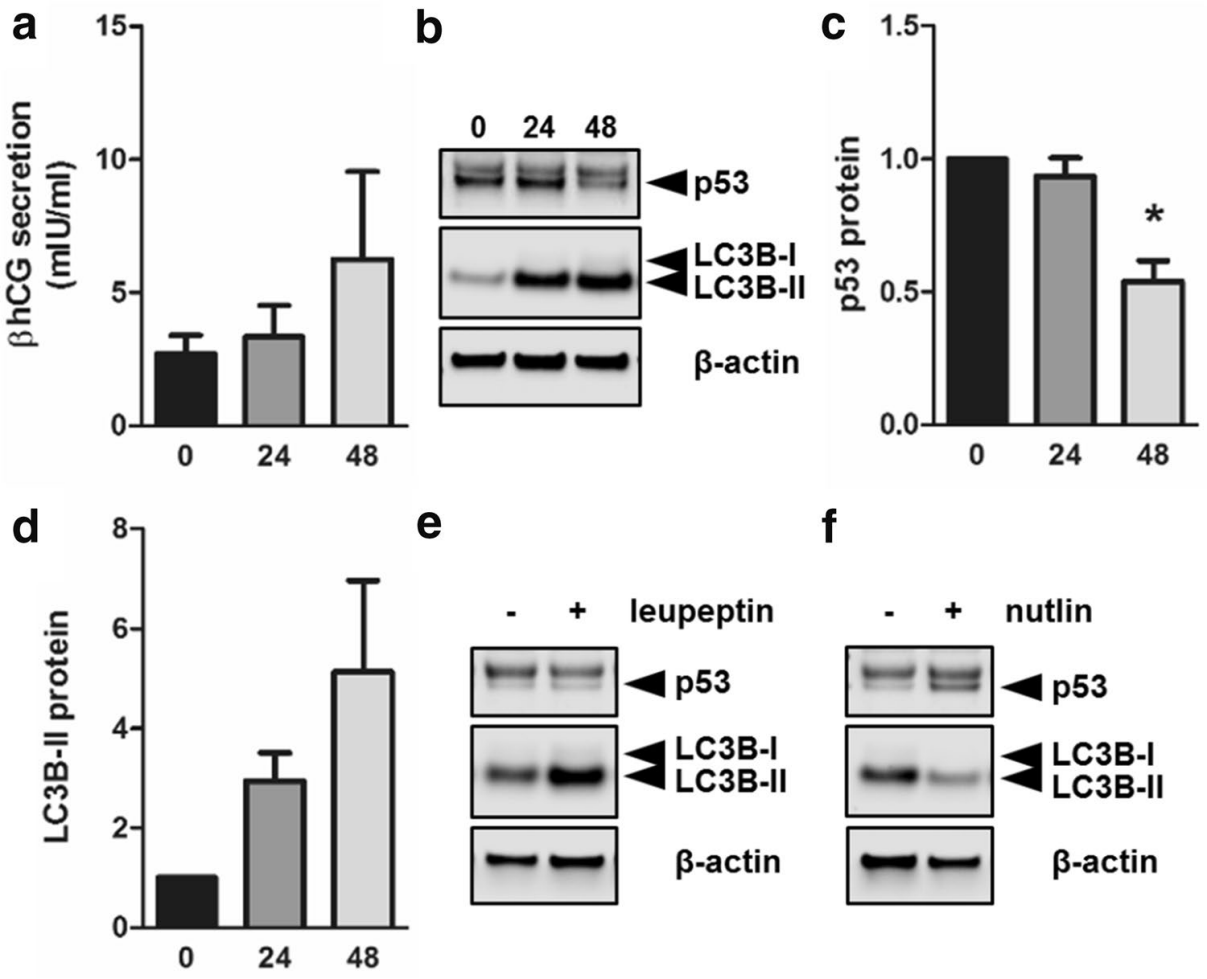
p53 and autophagic activity in primary trophoblast culture. Primary term trophoblasts were seeded 1 day prior to time point 0 h. Culture supernatants and cell lysates were collected at indicated time points. **a** Secretion of βhCG increased over time and suggested spontaneous differentiation of cultured trophoblasts. Immunoblotting (**b**) and band densitometry showed declining p53 levels (**c**), while LC3B-II levels (**d**) increased during culture. Incubation of trophoblasts with leupeptin (100 µM) did not change p53 protein, whereas LC3B-II levels (**e**) increased after 48 h incubation. Nutlin-3a treatment (10 µM, 48 h) increased p53 and decreased LC3B-II levels (**f**) when compared to control. Data in **a**, **c** and **d** are presented as mean ± SEM from three different trophoblast preparations. *p ≤ 0.05

To show that observed effects of p53 on autophagy in trophoblast differentiation are independent of forskolin treatment; levels of p53 and autophagy marker LC3B-II were analyzed in primary term trophoblast. Analysis of culture supernatants showed a time-dependent increase in βhCG secretion (Fig. 6a), confirming spontaneous differentiation of primary trophoblast. Immunoblotting for p53 protein revealed a double band. While the upper band remained largely unchanged in our experimental conditions, the lower declined during culture (Fig. 6b) and increased in presence of Nutlin-3a (Fig. 6f)—a compound that stabilizes p53 by competitively inhibiting its interaction with Mdm2, and thereby prevents its proteasomal degradation [52]. Thus, only the lower band was considered as specific for p53. Respective band densitometry showed that p53 levels significantly declined by 46% in primary trophoblasts after 48 h culture (Fig. 6b, c), while at the same time LC3B-II levels increased 5.1-fold (Fig. 6b, d). Further, leupeptin treatment substantially increased LC3B-II after 48 h (Fig. 6e), suggesting active autophagic flux in cultured primary trophoblasts. However, when primary trophoblasts were cultured in the presence of Nutlin-3a, increased p53 levels were accompanied by a remarkable decrease of LC3B-II after 48 h culture (Fig. 6f).

### Nutlin-mediated p53 stabilization decreased LC3B-II levels in placental explants

**Fig. 7:**
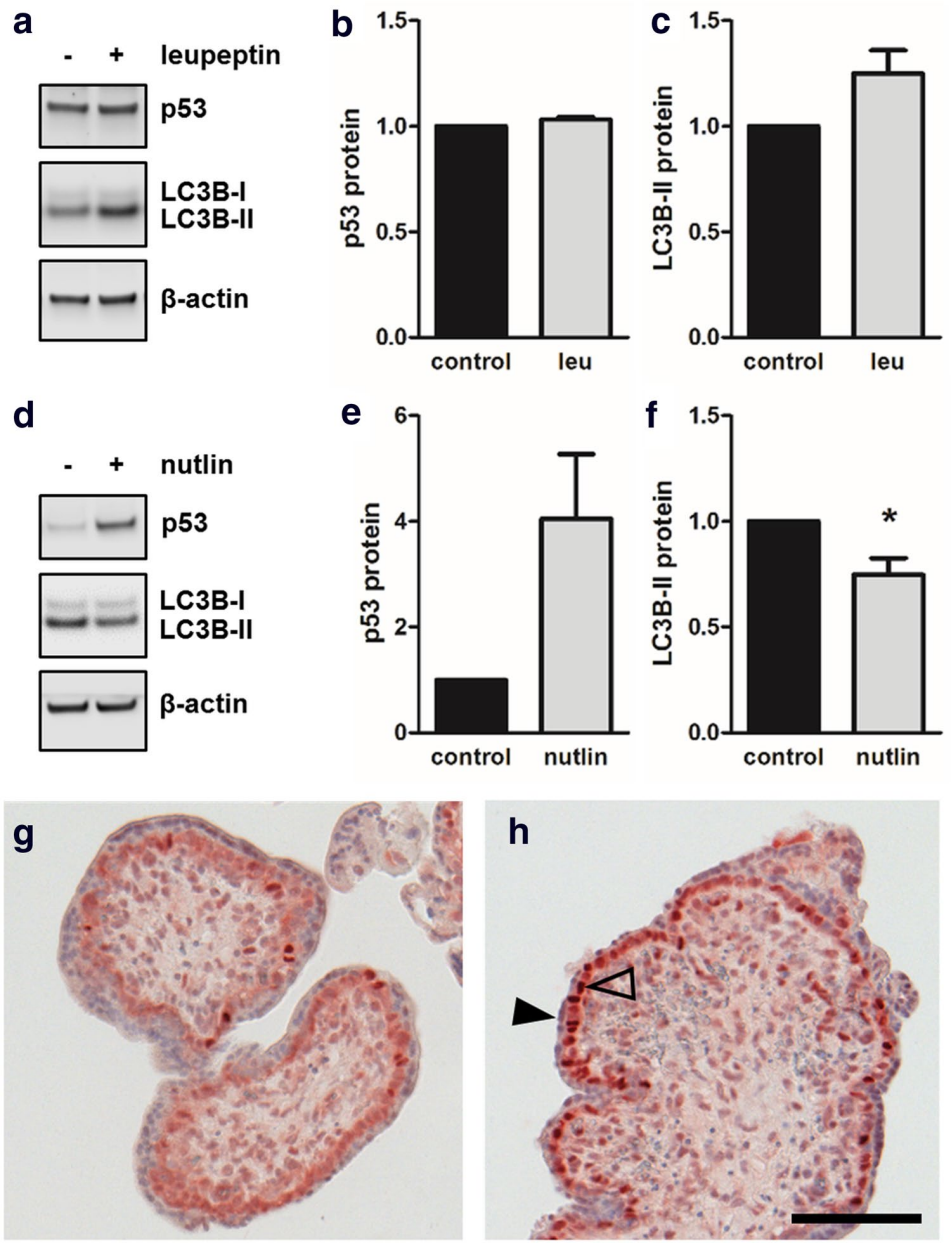
Nutlin-3a increased p53 and reduced LC3B-II levels in placental explants. Incubation of human first trimester placental explants with leupeptin (100 µM) and subsequent immunoblotting (**a**) showed unchanged p53 protein (**b**) and increased LC3B-II levels (**c**) after 48 h incubation, suggesting basal autophagic activity in placental explant culture. Nutlin-3a treatment (10 µM, 48 h) substantially increased placental p53 levels, whereas LC3B-II levels at the same time declined when compared to solvent control (**d**, **e**, **f**). Immunohistochemistry of control (**g**) and Nutlin-3a treated placental explants (**h**) revealed increased p53 staining intensity of villous cytotrophoblast nuclei (open arrowhead), while nuclei of the syncytiotrophoblast (arrowhead) remained unstained in response to Nutlin-3a. Scale bar in **h** represents 100 µm. Data in **b** and **c** are presented as mean ± SEM from three and those in **e** and **f** from four different patients. *p ≤ 0.05

Next, we compared data from BeWo cell model and primary trophoblasts to placental explant culture—an in vitro model which may be considered as more physiologic since villous trophoblasts remain in their natural microenvironment. For this purpose human first trimester placental explants were first incubated with or without leupeptin, and p53 as well as LC3B-II protein levels determined with immunoblotting. While p53 remained unchanged (Fig. 7a, b), LC3B-II levels showed a trend to increase in presence of leupeptin, which, however, did not reach statistical significance, when compared to control (Fig. 7a, c). Interestingly, levels of placental LC3B-II were consistently stronger than LC3B-I, irrespective of treatment. Together, these data suggested basal autophagic activity in cultured placental explants.

In contrast, incubation with Nutlin-3a showed 4.1-fold increased placental p53 levels, when compared to control (Fig. 7d, e). Stabilization of placental p53 levels by Nutlin-3a was accompanied by a 25.4% decrease in LC3B-II levels. Additionally, immunohistochemistry of placental explants showed increased p53 staining intensity of villous cytotrophoblast nuclei after incubation with Nutlin-3a (Fig. 7h), compared to control. Interestingly, nuclei of the syncytiotrophoblast remained unstained in response to Nutlin-3a, suggesting that p53 downregulation and/or proteasomal degradation may precede fusion and occurs already in the underlying cytotrophoblast.

### Forskolin had no effect on p53 and autophagy in non-fusing JAR trophoblasts

**Fig. 8:**
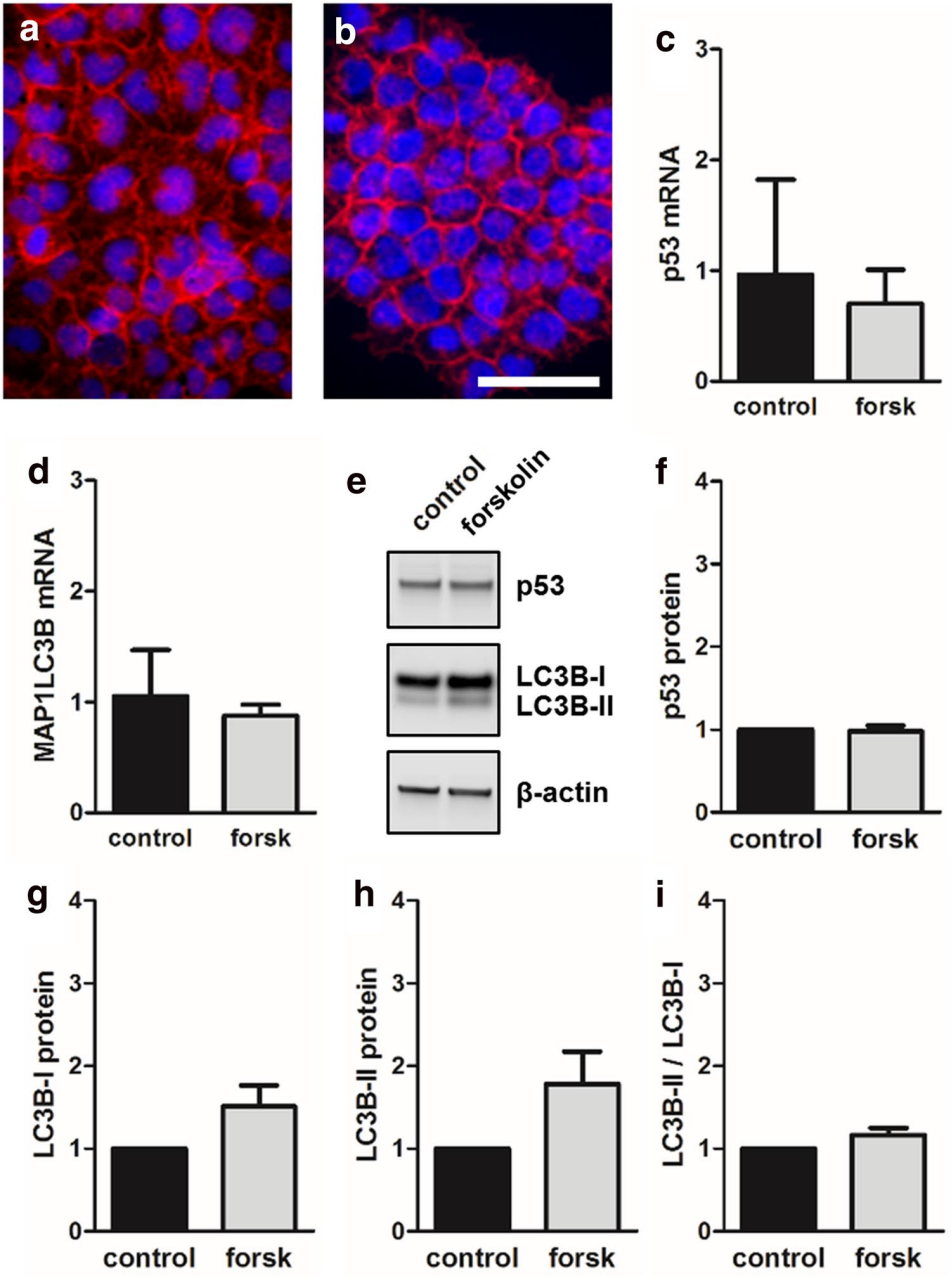
Effect of forskolin on p53 and autophagy in non-fusing JAR trophoblasts. Immunofluorescence staining of E-cadherin in JAR cells incubated either with vehicle control (**a**, DMSO, 0.1%) or forskolin (**b**, 20 µM) did not show formation of multinucleated syncytia after 48 h culture. qPCR analysis of p53 (**c**) and LC3B (**d**) mRNA as well as immunoblotting (**e**) with subsequent band densitometry for p53 (**f**), LC3B-I, and LC3B-II (**g**–**i**) protein levels did not show significant differences between forskolin (20 µM) treated JAR cells and vehicle control (DMSO, 0.1%) after 48 h. Scale bar in **b** represents 100 µm. Data in **c**, **d** and **f**–**i** are presented as mean ± SEM from three independent experiments, using different cell passages

Finally, effects observed for fusion-competent BeWo cells were compared to non-fusing trophoblast cell line JAR [6]. Immunofluorescence staining of JAR cells for transmembrane protein E-cadherin did not show formation of syncytia (i.e., multinucleated cells) in response to forskolin treatment after 48 h (Fig. 8a, b). In this cell line, forskolin stimulation had no significant effect on p53 and LC3B mRNA expression, respectively (Fig. 8c, d). In line with mRNA data, p53 protein levels did not change upon forskolin treatment (Fig. 8e, f). Analysis of LC3B protein showed stronger LC3B-I than LC3B-II levels, suggesting minimal autophagic activity in JAR cells under basal conditions (Fig. 8e). In the presence of forskolin, both LC3B-I and LC3B-II levels were slightly but not significantly increased, whereas the ratio of LC3B-II to LC3B-I remained unchanged (Fig. 8g–i).

## Discussion

Here, we provide evidence that p53 regulates autophagic activity in human villous trophoblasts. Once trophoblasts undergo differentiation and fuse with the multinucleated syncytium, p53 levels decline while autophagic activity increases. Activated autophagy during trophoblast differentiation is in agreement with a recent study by Cao et al. that shows increased levels of LC3B-II in forskolin-treated BeWo cells and suggests that autophagy levels positively correlate with syncytialization [8]. Our data show reciprocal levels of p53 and LC3B-II protein not only in forskolin-induced BeWo cells but also in primary trophoblasts undergoing spontaneous differentiation, suggesting a forskolin-independent effect. Most importantly, we show in BeWo cells, primary trophoblasts, and placental explants that preventing the decrease in p53 levels, via ectopic overexpression or pharmacological stabilization with Nutlin-3a, significantly reduces autophagy marker LC3B-II, indicating that p53 downregulation is a *conditio sine qua non* for functional autophagy in the syncytiotrophoblast. Consistent with our in vitro results, p53 is strongly reduced in the syncytiotrophoblast layer, while showing high levels of p53 staining in cytotrophoblast nuclei.

p53 has been attributed an important role in stem cell differentiation: human induced pluripotent stem cells (iPSCs) differentiate into several lineages with much higher efficiency when p53 is artificially downregulated via RNAi [25], presumably by alleviating p53’s cell cycle arrest and senescence activity [48]. In particular, p53 knockdown in iPSCs enhanced the rate of lineage conversion into trophoblast stem cells [27]. Furthermore, impaired repression of p53 levels in SENP2 knockout mice impedes trophoblast development [10]. However, in our study, overexpression of p53 during BeWo differentiation did not influence expression of differentiation markers and βhCG secretion, indicating that reduced p53 levels in the syncytiotrophoblast are a prerequisite for autophagy but not for differentiation per se. It is conceivable that the reports in iPSCs and in SENP2-knockout mice reflect p53’s action on lineage commitment very early in stem cell development, while BeWo cells and isolated primary trophoblasts are pre-committed cells with different specifics of p53 signaling.

Deletion, depletion, or inhibition of p53 has previously been associated with signs of induced autophagy, including conversion of LC3-I to LC3-II, redistribution of LC3B-GFP in cytoplasmic puncta and autophagosome formation [30]. Accordingly, colon cancer cells, human fibroblasts, and neuroblastoma cells show accumulation of autophagosomes and increased LC3B-II levels when p53 is silenced by siRNA, knocked out by homologous recombination, or inhibited by the p53 antagonist pifithrin-α [51]. We speculated about the consequence of p53 knockdown on autophagic activity during BeWo differentiation, but realized that cell seeding density required for siRNA transfection does not allow appropriate formation of syncytia. This obstacle has previously been shown for myocytes, which did not fuse into myotubes due to very low cell density [15].

Based on our results and our proposed role of p53 in repressing autophagy in villous trophoblasts, it is tempting to speculate about an association of abnormal p53 levels and dysregulated autophagy in placenta pathologies. Indeed, autophagy is described to occur in the syncytiotrophoblast of normal human placenta and is enhanced in placentas from pregnancies complicated by preeclampsia (PE) and/or intrauterine growth restriction (IUGR). Accordingly, immunoblotting for LC3B-II and electron microscopy indicate elevated autophagy levels in placentas from cases of PE [1, 24, 55] and IUGR [11, 22]. Although the available data for p53 in placental pathologies are inconsistent, they altogether suggest aberrant levels in pregnancies complicated by PE and IUGR. Previous expression analyses show significant higher p53 levels in placental tissue from IUGR cases [22, 42], in which most pronounced differences in p53 were observed in cytotrophoblast nuclei [28]. In severe early onset PE, p53 protein was shown to be significantly decreased, whereas no changes could be detected on mRNA level [42]. In contrast, other studies show significantly increased p53 protein in PE cases [37, 44], while another recent report shows decreased placental p53 mRNA levels in PE, PE associated with IUGR and in cases with IUGR only [13]. These inconsistencies may be explained by low case numbers, differences in gestational age at delivery, and different severity of pathology and call for further studies.

The dominant mechanism for regulating wild-type p53 at the post-transcriptional level is via its interaction with the endogenous inhibitor Mdm2 [32], an E3-ubiquitin ligase known to ubiquitinate p53 to target it for proteasomal degradation. Thus, aberrant placental p53 protein levels, with unchanged p53 mRNA expression as described by some studies for PE and IUGR cases, can be explained by regulation of p53 protein stability via Mdm2 or similar E3 ubiquitin ligases. Since p53 is mainly regulated post-transcriptionally in most cells, much less is known about mechanisms of p53 regulation on the transcriptional level. However, in different cell systems, p53 mRNA levels have been shown to be regulated by adjusting the rate of transcription (histone deacetylases (HDAC9, [59]), by modulation of mRNA stability (i.e. via miRNA-1228 [57]) or by hybridization of antisense transcript Wrap53 [29]. Also, in the p53 promoter, response elements of several transcription factors [43], [e.g., cAMP-responsive element-binding protein (CREB [38])], have been identified. Furthermore, in JAR trophoblast cells, p53 was shown to bind to the MMP-2 promoter in a cAMP-responsive manner [49]. In contrast, in our study, we find p53 downregulation upon treatment with the cAMP-elevating reagent forskolin and in primary trophoblasts undergoing spontaneous differentiation. These data indicate a novel, cAMP-independent way of p53 mRNA repression in trophoblasts.

The exact role of autophagy in villous trophoblast homeostasis is unclear. Several previous electron microscopy studies show autophagic vacuoles predominantly in the syncytiotrophoblast of normal placenta, suggesting autophagy to be a constitutive process, which may ensure survival in times of moderate nutrient depletion and/or oxidative stress [11]. In this context, a study by Broad and Keverne suggests that placental autophagy—mainly ribophagy—provides nutrients that can be mobilized to protect fetal growth and brain development, in situations of acute deprivation [7]. Besides ensuring placental and fetal nutrition, autophagy may represent a pivotal mechanism for organelle reorganization required after incorporation of cytoplasmic contents of the fusing cytotrophoblast into the syncytium. This assumption is based on observations in myotube formation, showing dynamic remodeling of the mitochondrial network, including mitochondrial clearance and biogenesis [15]. Importantly, this mitochondrial remodeling is impaired when autophagy is inhibited [45]. In the human placenta, mitochondria of the syncytiotrophoblast show a different appearance, compared to cytotrophoblasts [4]. Thus, autophagy-dependent remodeling of the mitochondrial network, as described for myotube formation, may well take place in the syncytioplasm in areas of freshly incorporated cytotrophoblasts. Our immunofluorescent and electron microscopy images show a distinct location in apical cytoplasm of autophagy markers and autophagic compartments in the syncytiotrophoblast layer in first trimester placentas. Because of this specific proximity to the maternal circulation, it suggests that autophagy might be involved in fetal-maternal communication and/or transport. However, if these structures are exocytosed to release its content into maternal circulation or only used for intrasyncytial turnover remains unclear and needs further investigation.

In summary, we show that autophagic activity and p53 levels are reciprocally regulated in villous cytotrophoblasts and the syncytiotrophoblast. Furthermore, artificially maintaining p53 levels reduces autophagy marker LC3B-II. Thus, our study suggests that differentiation-dependent downregulation of p53 is a prerequisite for activating autophagy in the syncytiotrophoblast. Activated autophagy may account for organelle and cytoskeleton rearrangement during transition from mononucleated into multinucleated trophoblast, thereby providing recycled components to the syncytiotrophoblast.

## Materials and methods

### Human placenta tissue samples

The study was approved by the ethical committee of the Medical University of Graz. First trimester placental tissues were obtained between weeks 6 and 12 of gestation with written informed consent from women undergoing legal elective pregnancy terminations. Term placenta tissues were obtained from uncomplicated pregnancies after delivery between weeks 38 and 40.

### Culture of trophoblast cell lines

BeWo and JAR cells were purchased from the European Collection of Cell Cultures (ECACC) and were cultured as previously described [18]. In brief, BeWo were cultured in DMEM/F12 (1:1, Gibco, life technologies; Paisley, UK) supplemented with 10% FCS (Gibco), penicillin/streptomycin (Gibco), amphotericin B (Gibco) and l-glutamine (Gibco) in a humidified atmosphere of 5% CO_2_ at 37 °C. JAR cells were cultured in RPMI medium (Gibco) including same supplements and culture conditions as described for BeWo. Cells between passage 10 and 20 were used for in vitro experiments. In case of forskolin treatment, culture medium was supplemented with forskolin (Tocris, Bio-techne, Abingdon, UK) at a final concentration of 20 µM (20 mM stock in DMSO). Control cells were incubated with culture medium containing the same volume of DMSO (0.1%).

For leupeptin treatments, BeWo cells (2 × 10^5^ cells/well) were plated in 12-well dishes (Nunc Lab-Tek, Thermo Fisher; NY, USA) in above described culture medium 1 day prior experimental start. Next day, cells were incubated in the presence or absence of forskolin (20 µM) for 48 h at 37 °C. Leupeptin (Sigma-Aldrich, St. Louis, MO, USA) was added to cultures 24 h prior to the end of experiments at a concentration of 100 µM. DMSO (0.1%) and aqua dest were used as vehicle controls for forskolin and leupeptin, respectively.

### Isolation and culture of primary term trophoblasts

Primary trophoblasts were isolated from chorionic villi of three term placentas with informed consent from women with uncomplicated pregnancies and approval by the ethical committee of the Medical University of Graz. Isolation was performed by enzymatic digestion and Percoll density gradient centrifugation as described previously [5]. Trophoblasts (3 × 10^6^ cells/well in six well dishes) were cultured in DMEM (Gibco, lifetechnologies) with 10% FCS (v/v), 100 mg/ml streptomycin and 100 IU/ml penicillin (Gibco, lifetechnologies). A representative proportion of primary cells were scrutinized for purity by immunocytochemistry [5] and viability/differentiation was monitored by measurements of secreted human chorionic gonadotropin beta-subunit (βhCG).

### LC3B-GFP experiments

BeWo cells (1.2 × 10^5^ cells/well) were seeded in chamber slides (Nunc) 1 day prior experimental start. Next day, cells were transfected with 0.5 µg LC3B-GFP vector using K2 Transfection System (Biontex Laboratories GmbH, Munich, Germany), according to the manufacturer’s protocol. After 24 h, medium was changed and cells were stimulated with forskolin (20 µM) or vehicle control DMSO (0.1%) for 48 h at 37 °C. Leupeptin (100 µM) was added to cultures for the last 4 h of experiments. For live cell imaging of LC3B-GFP puncta, cells were washed with PBS and nuclei stained with Hoechst 33342 (1:10,000 diluted, Thermo Fisher) for 10 min at 37 °C. Twenty randomly selected fields per condition were microphotographed (40× objective) using Zeiss Observer Z1 inverted microscope (Carl Zeiss, Oberkochen, Germany) and the ZEN 2.3 software (Carl Zeiss, blue edition, Version 2.3.64.0). Quantification of GFP-positive puncta related to DAPI signal within an image frame was performed using Ilastik Interactive Learning and Segmentation software followed by pixel counting with an R script as recently described [40].

### p53 overexpression experiments

BeWo cells (3 × 10^5^ cells/well) were seeded in 12 well dishes in above described culture medium 1 day prior experimental start. Next day, cells were transfected with either p53 vector (#10838, 1 µg/well, Addgene; Cambridge, MA, USA) or empty vector control using K2 Transfection System (Biontex Laboratories GmbH, Munich, Germany), according to the manufacturer’s protocol. After 24 h, medium was changed and cells were stimulated with forskolin (20 µM) or vehicle control DMSO (0.1%) for 48 h. Leupeptin (100 µM) was added to cultures 24 h prior to the end of experiments.

### Measurement of secreted βhCG

Culture media were collected at indicated time points and centrifuged at 4000 rpm for 10 min. Supernatants were stored at − 20 °C and subjected in groups to routine immunoassay analyses (Dimension Xpand; Dade Behring Inc., Deerfield, Illinois).

### Placental explant culture

Placental villous tissue from human first trimester was thoroughly rinsed in buffered saline and dissected into small pieces of approximately 5 mg moist mass as described previously [46]. Placental explants were cultured in six well dishes (Nunc) and 4 ml/well DMEM/F12 supplemented with 10% FCS, penicillin/streptomycin, amphotericin B and l-glutamine in a hypoxic workstation (BioSpherix; Redfield, NY, USA) under 2.5% oxygen for 48 h at 37 °C. For treatments, culture medium was supplemented with Nutlin-3a (Cayman chemical, USA) at a working concentration of 10 µM. DMSO at equal volume was used as vehicle control.

### Microarray analyses

Applied Biosystems Human Genome Survey Arrays (Applied Biosystems, Foster City, CA, USA) were used to determine the transcriptional profiles of BeWo cells treated with forskolin (20 µM, 48 h) or DMSO (0.1%, 48 h) as previously described [18]. Briefly, digoxigenin-UTP labeled cRNA was generated and linearly amplified from 30 μg of total RNA using Applied Biosystems Chemiluminescent RT-IVT Labeling Kit. cRNA was fragmented and hybridized to the Applied Biosystems Human Genome Survey Microarray V2.0 (29,098 genes) and imaged on an AB1700 chemiluminescent microarray analyzer. Gene expression profiles are derived from the microarray raw data by quantile normalization and averaging of each three replicates. The data set was submitted to NCBI gene expression omnibus (Accession #: GSE98523).

Genes differentially up- or downregulated more than twofold were submitted separately to DAVID functional annotation web tool or to ingenuity pathway analysis (IPA) to identify upstream regulators [40].

### qPCR analysis

Total RNA was isolated with peqGOLD TriFast (VWR, Radnor, Pennsylvania, USA) according to the manufacturer’s instructions. Quality check was followed by reverse transcription of 1 µg total RNA per reaction using High-Capacity cDNA Reverse Transcription Kit (Applied Biosystems) according to manufacturer’s manual. qPCR was performed with Universal SYBR Green Supermix (Bio-Rad, Hercules, CA, USA) using a Bio-Rad CFX96 cycler and specific primers for *MAP1LC3B* (MAP1LC3B_For: AAGGCGCTTACAGCTCAATG and MAP1LC3B_Rev: CTGGGAGGCATAGACCATGT) and *TP53* (TP53_For: CAGCACATGACGGAGGTTGT and TP53_Rev: TCATCCAAATACTCCACACGC) as described previously [39, 40]. Moreover, primers for GCM1 (GCM1_For: TTCCCGGTCACCAACTTCTG and GCM1_Rev: GTAAACTCCCCTGACTTTGTGTT), syncytin-1 (ERVW-1_For: CCATGCCGCTGTATGACCAG and ERVW-1_Rev: GGGTTCCCTTAGAAAGACTCCT), syncytin-2 (ERVFRD-1_For: ACCGCCATCCTGATTTCCC and ERVFRD-1_Rev: GAGGCTGGATAAGCTGTCCC), p62 (SQSTM1_For: GACTACGACTTGTGTAGCGTC and SQSTM1_Rev: AGTGTCCGTGTTTCACCTTCC) and hCG beta-subunit (CGB_For: TGAGCCACTCCTGCGCCC and CGB_Rev: CAGCCCCTGGAACATCTCCA) were used. Ct values and relative quantification of gene expression were automatically generated by the CFX Manager 2.0 Software (Bio-Rad) using the expression of *GAPDH* (GAPDH_For: ACCCACTCCTCCACCTTTGA and GAPDH_Rev: CTGTTGCTGTAGCCAAATTCGT) as reference.

### Immunoblotting

After incubations, cells and placental explants were washed with PBS and homogenized in RIPA buffer (Sigma-Aldrich) including protease inhibitor cocktail (Roche Diagnostics; Mannheim, Germany). Homogenates were centrifuged at 8000×*g* and 4 °C for 10 min. Concentration of total tissue protein was determined in clear supernatants according to Lowry method. 30 µg of total protein were applied to precast 10% Bis–Tris gels (NuPAGE, Novex; lifetechnologies). Proteins were blotted on a 0.45-µm nitrocellulose membrane (Hybond, Amersham Biosciences, GE Healthcare Life Sciences, Little Chalfont, UK) and blotting efficiency determined with Ponceau staining (Ponceau S solution, Sigma-Aldrich). Membranes were cut in horizontal strips at molecular weight ranges for target proteins. Mouse monoclonal anti-p53 antibody (1:2000, clone DO-1, Santa Cruz, Dallas, TX, USA), polyclonal LC3B/MAP1LC3B antibody (1:1000, NB100-2220, novusbio, Bio-techne), polyclonal anti-p62/SQSTM1 antibody (1:1000, #P0067, Sigma-Aldrich) and monoclonal anti-beta actin antibody (1:20,000; clone AC-15, abcam, Cambridge, UK) were applied to membrane strips overnight at 4 °C. HRP conjugated goat anti-mouse and goat anti-rabbit IgG (1:3000, Bio-Rad), respectively, were used as secondary antibodies and incubated on membranes for 2 h at RT. Immunodetection was performed with a chemiluminescent immunodetection kit (Amersham ECL Prime Western blotting detection Reagent) according to the manufacturer´s instructions. Images were acquired with FluorChem Q System (Alpha Innotech, Cell Bioscienes, Santa Clara, CA, USA) and band densities were analyzed with Li-Cor Image Studio Lite 5.2. Results are presented as a ratio of target protein and beta-actin band densities.

### Immunofluorescence

Morphological evaluation of syncytialization was performed by staining transmembrane protein E-cadherin to visualize cell borders. For this purpose, BeWo and JAR cells (8 × 10^4^ cells/chamber) were seeded in chamber slides and incubated with either forskolin (20 µM) or vehicle control (DMSO, 0.1%) for 48 h. Thereafter, cells were washed with PBS, dried and fixed in acetone for 10 min. Rehydration with PBS was followed by incubation with Ultra Vision Protein-Block (LabVision, Thermo Fisher Scientific, Runcorn, UK) for 7 min. Monoclonal anti-E-Cadherin antibody (Acris Antibodies GmbH, OriGene EU, Herford, Germany) was diluted 1:15 in antibody diluent (DAKO, Carpintera, CA, USA) and incubated on cells for 30 min. PBS washing steps were followed by incubation with secondary antibody, Alexa Fluor 555 goat anti-mouse (1:200; Invitrogen, Lifetechnologies, Carlsbad, CA, USA) for 30 min. Slides were washed and nuclei were stained with DAPI (1:2000; Invitrogen) for 5 min.

For p53 and LC3B double fluorescence, formalin fixed and paraffin embedded (FFPE) human first trimester (*n* = 3) was cut in 5 µm sections and mounted on Superfrost Plus slides (Menzel/Thermo Fisher Scientific; Braunschweig, Germany). Standard deparaffinization was followed by boiling sections in 10 mM sodium citrate buffer (pH 6.0) for antigen retrieval. Slides were incubated with anti-p53 antibody (1:50, clone DO-X, Santa Cruz) and anti-LC3 antibody (1:5000, NB100-2220, novusbio) for 45 min. Subsequently, slides were washed and incubated with a mixture of Alexa Fluor 555 goat anti-rabbit and Alexa Fluor 488 goat anti-mouse antibodies (both 1:200, Invitrogen) for 30 min at room temperature. Slides were washed and nuclei were stained with DAPI (1:2000; Invitrogen). For negative controls, negative control for rabbit IgG (Ab-1, Thermos Fisher Scientific) and negative control mouse IgG2a (DAKO) were used and generated no staining. After staining, cells as well as tissue sections were mounted with ProLong Gold antifade reagent (Invitrogen). Fluorescence microscopy was performed using a Zeiss Observer Z1 inverted microscope (Carl Zeiss, Oberkochen, Germany).

### Immunohistochemistry and immunocytochemistry

Human first trimester (*n* = 5) and term FFPE placental tissue (*n* = 5) sections (5 μm) were mounted on Superfrost Plus slides. After deparaffinization slides were subjected to antigen retrieval by boiling sections in 10 mM sodium citrate buffer (pH 6.0) for 7 min at 120 °C. Sections were stained using the UltraVision Large Volume Detection System HRP Polymer Kit (Thermo Fisher Scientific) as previously described [47]. In brief, endogenous peroxidase was blocked with UltraVision hydrogen peroxide block for 10 min. Three washing steps with TBS including 0.05% Tween 20 (TBS/T; Merck; Darmstadt, Germany) were followed by Ultra Vision Protein Block for 5 min. Monoclonal anti-p53 antibody (1:50, clone DO-1, Santa Cruz) was diluted in Antibody Diluent (DAKO) and incubated on slides for 45 min at RT. After three TBS/T washing steps detection was achieved by incubation with primary antibody enhancer and a HRP-labeled polymer system (15 min) and 3-amino-9-ethylcarbazole (AEC, Thermo Scientific), according to the manufacturer’s instructions. Nuclei were stained with hemalaun and slides were mounted with Kaiser’s glycerol gelatine (Merck). P62 staining was performed as described above, using antigen retrieval buffer pH 9.0 and polyclonal anti-p62/SQSTM1 antibody (1:10,000, Sigma-Aldrich).

For immunocytochemistry, BeWo cells were seeded in chamber slides (8 × 10^4^/chamber) and were stimulated with forskolin (20 µM) or vehicle control DMSO (0.1%) for 48 h. After culture, cells were washed with PBS, dried and fixed in acetone for 10 min. Rehydration with PBS was followed by p53 staining procedure as described for immunohistochemistry, except antigen retrieval procedure. For negative control, slides were incubated with negative control mouse IgG2a at the same concentration as for anti-p53 antibody. Images were acquired with a Leica microscope (Leica DM6000B) and a digital camera (Olympus DP72).

### Transmission electron microscopy

For transmission electron microscopy, first trimester placenta was fixed in 0.1 M phosphate buffer (pH 7.4) containing 2.5% glutaraldehyde and 2% formaldehyde (2 h), post-fixed in 2% OsO4 (2 h), dehydrated in graded series of ethanol, and embedded in a TAAB epoxy resin (Gröpl). Ultrathin sections (75 nm) were cut with a Leica UC 7 and stained with lead citrate and platine blue. Images were taken on a FEI Tecnai G2 20 transmission electron microscope (FEI, Eindhoven, the Netherlands) with a Gatan ultrascan 1000 CCD camera (acceleration voltage 120 kV.

### Quantification of p53 immunostaining in BeWo cells

Imaging was performed on a Leica Microscope with 20× magnification and 24 or 32 randomly selected fields per sample. Quantification of immunostaining was performed with the open-source cell image analysis software CellProfiler (Version 2.1.1) [9]. The pipeline was designed for quantification of AEC staining within the cell nucleus. In short, images were processed with the “Identify Primary Objects” modules to identify cell nuclei and AEC stained regions. Nuclei areas were applied as mask using the “Mask Image” module, thereby defining a positive region for quantification of AEC staining. We used the “Measure Image Intensity” module to quantify AEC intensity with size threshold of 5–1000 pixels with lower and upper intensity threshold of 0.6–1.0. For each image, total AEC pixel intensity values were divided through the nuclear pixel area.

### Statistical analysis

Data were analyzed using SigmaPlot 12.5 and are presented as mean ± SEM. Data were subjected to normality test (Shapiro–Wilk test) and equal variance test. In case of normally distributed data differences between groups were tested using two-tailed *t* test. Otherwise, Mann–Whitney rank sum test was applied. For multiple comparison procedure, one-way repeated measures analysis of variance was followed by Holm–Sidak method to isolate groups that differ from the others. One sample *t* test was used when controls were set as 1. A *p* value of less than 0.05 was considered statistically significant.
